# Postoperative muscle mass restoration as a prognostic factor in patients with resected pancreatic cancer

**DOI:** 10.1371/journal.pone.0238649

**Published:** 2020-09-16

**Authors:** Jongchan Lee, Jong-chan Lee, Hyoung Woo Kim, Jaihwan Kim, Jin-Hyeok Hwang

**Affiliations:** 1 Department of Internal Medicine, College of Medicine, Seoul National University Bundang Hospital, Seongnam, Korea; 2 Department of Internal Medicine, College of Medicine, Chungbuk National University Hospital, Cheongju, Korea; University of Nebraska Medical Center, UNITED STATES

## Abstract

**Background:**

Recent studies have found that muscle depletion may be a prognostic predictor in patients with pancreatic cancer (PC). However, in these studies, limited data were used to assess the relationship between the serial change in body composition and outcomes after PC resection. Hence, we evaluated the changes in body composition during the perioperative period in patients with PC and their association with the overall survival (OS).

**Methods:**

A total of 89 patients with PC who received surgery with curative intent between 2006 and 2015 were included in this study retrospectively. These patients underwent serial computed tomography (CT) scans: preoperatively, immediately after surgery (4 weeks), and 12 and 24 weeks after resection. The muscle and visceral fat areas were measured at the third lumbar vertebra level on cross-sectional CT images using sliceOmatic V5.0 program (TomoVision, Canada). The body composition ratio was determined by dividing the post-resection body composition at each point (4, 12, and 24 weeks) by the pre-resection body composition. Patients were divided into two groups—higher and lower groups—based on this body composition ratio (skeletal muscle mass ratio [SMR], visceral fat mass ratio [VFR]). The OS was compared between the two groups using the log-rank test.

**Results:**

The median age of patients was 63 (27–84) years, and the baseline body mass index was 23.0 (17.0–35.8) kg/m^2^. In the comparison of the SMR, there was no significant difference in the OS between the two groups at 4 and 12 weeks (4 weeks, *P* = 0.488; 12 weeks, *P* = 0.397). However, the higher group showed a longer OS than the lower group at 24 weeks (39 vs. 20 months, *P* = 0.008). Similarly, in the VFR, there was no significant difference in the OS between the two groups at 4 and 12 weeks (4 weeks, *P* = 0.732; 12 weeks, *P* = 0.060). However, the OS was longer in the higher group at 24 weeks (35 vs. 22 months, *P* = 0.023). When we analyzed the effect of muscle restoration at 24 weeks after resection on the OS by gender, there was no significant difference between the OS and SMR in the male group (*P* = 0.213), but a significant difference was noted in the female group (*P* = 0.002). In the multivariate Cox regression analysis, the SMR at 24 weeks after resection was significantly associated with the OS (*P* = 0.023) but not VFR at 24 weeks. In 69 patients without recurrence at 6 months, the SMR at 24 weeks was related to longer OS but without statistical significance (*P* = 0.07).

**Conclusions:**

This study suggests that the restoration of muscle mass at 24 weeks after resection may be an independent prognostic factor for survival in patients with resected PC.

## Introduction

Despite much advancement in the treatment of pancreatic cancer (PC), the prognosis remains poor. Adjuvant chemotherapy after surgical resection has been reported to improve the postoperative prognosis in patients with PC [1, 2]. Nonetheless, several patients still develop recurrence after curative resection [3, 4]. PC is one of the leading causes of cancer-related mortality worldwide [5]. Therefore, several studies are underway to investigate the potential factors influencing the prognosis of PC after resection [6, 7].

Sarcopenia is a condition that is characterized by the loss of muscle mass, strength, and function [8, 9]. Decreased muscle mass results in functional impairment, such as falls and loss of autonomy [10]. Several mechanisms are involved in the development of sarcopenia: age, endocrine, muscle disuse, inadequate nutrition, neurodegenerative disease, and cachexia [11]. Sarcopenia is increasingly recognized as an important issue even in cancer management. Most patients with cancer are exposed to several factors that cause muscle mass to decrease and muscle dysfunction, including malnutrition, physical inactivity, tumor-derived factors, and cancer therapy [12].

Several studies have been conducted to evaluate how the change in body composition influences the prognosis of patients with lung, esophageal, and colorectal cancers [13–15]; to our knowledge, such studies are currently underway for patients with PC [16–18]. PC is associated more with weight loss than other cancers because it can cause diabetes mellitus, gastrointestinal obstruction, and secretory disorders of digestive enzymes. Sarcopenia, sarcopenic obesity, and a decrease in muscle density on computed tomography (CT) at the time of diagnosis have been found to be associated with poor prognosis [6, 16, 19]. Moreover, a recent study has shown that a decrease in the skeletal muscle in patients with inoperable locally advanced PC is associated with poorer prognosis [20].

However, to date, there are only a few studies investigating the association between serial body composition change and prognosis over time in patients with resected PC. Hence, the purpose of this study was to investigate the trajectory of body composition after PC resection and the relationship between body composition change and overall survival (OS).

## Materials and methods

### Study patients

A total of 326 patients with PC who received resection at Seoul National University Bundang Hospital between January 2006 and December 2015 were reviewed. Among these, those who underwent surgery for palliation or received neoadjuvant treatment were excluded. Patients without follow-up CT scans available on a regular basis for up to 6 months after surgery were also excluded. Finally, 89 patients with PC were enrolled.

CT was performed at the time of diagnosis, immediately after resection (4 weeks), and at approximately 12 and 24 weeks after resection. CT scans using a pancreatic protocol were obtained to investigate any complications immediately after resection and again at 12 and 24 weeks after resection to assess possible recurrence or progression of the disease.

The medical records of all participants were collected and anonymized, and this study was approved by the institutional review board (IRB) of Seoul National University Bundang Hospital (Seongnam, Republic of Korea) (B-1912-585-104). Since this study was a retrospective analysis of clinical data, consent requirement was waived by the local IRB.

### Body composition measurements and definition of the changes in body composition

Images of the cross-sectional areas of the skeletal muscle mass (SMM) and visceral fat mass (VFM) at the midpoint of the third lumbar vertebral body (L3) were obtained via a CT scan. The cross-sectional image of the L3 level contained the psoas, erector spinae, quadratus lumborum, transversus abdominis, external and internal oblique muscles, and rectus abdominis. We analyzed these body compositions using the sliceOmatic V5.0 software (TomoVision, Magog, Canada). In brief, sliceOmatic is a program that makes it possible to distinguish specific tissues using the Hounsfield unit (HU) thresholds. The skeletal muscles were quantified between the HU range of -29 to 150 HU and the visceral adipose tissue using the range of -150 to -50 HU. This program summed the pixels of the area of each tissue and expressed it as a number. The boundary of the tissue was calibrated manually as needed. To prevent disagreement between the observers, one well-trained observer analyzed all 356 CT images using this program.

To determine the changes in the body composition during the follow-up period, we calculated the body composition ratio at each time point (4, 12 and 24 weeks after resection) to the body composition at the time of diagnosis: the skeletal muscle mass ratio (SMR) and visceral fat mass ratio (VFR). Then, we obtained the median values of SMR and VFR at each time point. Based on this, patients were divided into one of two groups at each time point: lower and higher group. We then compared the OS of two groups according to the body composition ratio.

### Statistical analysis

The patients’ baseline characteristics and body composition were presented as the mean and standard deviation for continuous variables and proportions and percentages for categorical variables. The survival time was defined as the time from diagnosis to death, and the median OS was determined by the Kaplan–Meier method. A comparison of survival of subgroups was performed using the log-rank test. The Cox proportional hazards regression model was used to determine the relationship between the variables and survival as hazard ratios (HR) and 95% confidence intervals (CI). The results with two-sided t-test and a value of *P* < 0.05 were considered to indicate statistical significance. Statistical analyses were performed using the STATA version 14.0 (StataCorp, College Station, TX, USA).

## Results

### Patient characteristics

This study included 89 patients with resected PC with four serial CTs before resection and 4, 12, and 24 weeks after resection. Their demographics and clinical characteristics are shown in Table 1. The median age was 63 years (range, 27–84 years), and 55 of 89 patients (61.8%) were men. The median body mass index (BMI) was 23.0 kg/m^2^ (range, 17.0–35.8 kg/m^2^). Thirty-eight patients (42.7%) had a history of preoperative diabetes mellitus. Approximately two-thirds of patients (69.7%) underwent pylorus-preserving pancreaticoduodenectomy (PPPD) or standard pancreaticoduodenectomy (PD), and the remainder underwent distal pancreatectomy (DP). R0 resection was achieved in 67 (75.3%) patients, and 57 (64.0%) patients received more than four cycles of effective adjuvant chemotherapy.

**Table 1:** Patient demographics and clinical characteristics.

Variables	*n* = 89
Age (range), years	63 (27–84)
Sex	
Male (%)	55 (61.8%)
Female (%)	34 (38.2%)
BMI* (range) (kg/m^2^)	23.0 (17.0–35.8)
Diabetes mellitus ASA grade†	38 (42.7%)
I	25 (28.1%)
II	54 (60.7%)
III	10 (11.2%)
Tumor location	
Head	57 (64.0%)
Body	15 (16.9%)
Tail	17 (19.1%)
Differentiation of tumor	
Well differentiated	7 (7.4%)
Moderately differentiated	71 (77.7%)
Poorly differentiated	6 (7.4%)
Undifferentiated	5 (7.4%)
Postoperative TNM stage	
IA	10 (11.2%)
IB	29 (32.6%)
IIA	9 (10.1%)
IIB	34 (38.2%)
III	7 (7.9%)
Type of resection	
Standard pancreaticoduodenectomy	16 (18.0%)
Pylorus-preserved pancreaticoduodenectomy	46 (51.7%)
Distal pancreatectomy	27 (30.3%)
Surgical margin state	
R0	67 (75.3%)
R1	22 (24.7%)
Adjuvant chemotherapy (≥4 cycles)	57 (64.0%)

* BMI, body mass index;

^†^ ASA grade, American Society of Anesthesiologists grade.

### Changes in body composition parameters

We investigated the change of trajectory of body compositions, such as BMI, SMM, and VFM (Table 2). The overall observation showed that the BMI, SMM, and VFM decreased after PC resection throughout the 12-week period after resection, regardless of gender. In men, the SMR and VFR decreased throughout the 24-week period after resection although the degree of reduction was lower. In women, however, the skeletal muscle and visceral fat reached nadir at 12 weeks, which then slightly recovered at 24 weeks (Table 2).

**Table 2:** Change in body composition parameters after resection of pancreatic cancer (mean, standard deviation).

Variables	Before resection	<4 weeks after resection	12 weeks after resection	24 weeks after resection
**Total**				
BMI	23.0 (19.0–26.0)	22.6 (18.1–27.1)	21.2 (17.0–25.4)	20.6 (15.8–25.4)
Skeletal muscle*, cm^2^	118.7 (93.4–144.1)	121.5 (98.7–144.2)	110.2 (85.5–135.0)	109.9 (86.5–133.3)
Skeletal muscle index†	44.7 (37.7–51.7)	45.9 (39.5–52.2)	41.5 (34.3–48.6)	41.5 (34.3–48.6)
Visceral fat§, cm^2^	94.6 (46.8–142.4)	86.6 (41.4–131.8)	64.9 (30.4–99.4)	62.3 (27.5–97.1)
Skeletal muscle ratio	1.000	1.031 (0.959–1.104)	0.931 (0.858–1.004)	0.931 (0.838–1.023)
Visceral fat ratio	1.000	0.943 (0.721–1.165)	0.767 (0.331–1.203)	0.758 (0.288–1.228)
**Males**				
BMI	22.5 (19.7–25.3)	21.8 (16.7–26.9)	21.1 (17.3–24.9)	20.8 (17.0–24.6)
Skeletal muscle, cm^2^	132.2 (111.3–153.2)	132.2 (111.9–152.5)	123.0 (101.9–144.1)	120.9 (99.7–142.0)
Skeletal muscle index	47.2 (40.5–53.9)	47.2 (40.8–53.7)	43.9 (37.2–50.6)	43.2 (35.8–50.6)
Visceral fat, cm^2^	100.5 (49.3–151.6)	90.6 (43.7–137.5)	68.6 (31.0–106.3)	65.9 (28.3–103.6)
Skeletal muscle ratio	1.000	1.002 (0.949–1.055)	0.934 (0.866–1.002)	0.916 (0.825–1.007)
Visceral fat ratio	1.000	0.925 (0.722–1.128)	0.749 (0.309–1.190)	0.735 (0.276–1.194)
**Females**				
BMI	23.7 (20.5–26.9)	23.7 (20.6–26.8)	21.4 (16.6–26.2)	20.4 (14.2–26.6)
Skeletal muscle, cm^2^	96.9 (82.8–111.0)	104.1 (89.9–118.3)	89.6 (75.7–103.4)	92.2 (77.8–106.6)
Skeletal muscle index	40.6 (35.1–46.1)	43.6 (38.1–49.2)	37.5 (32.0–43.1)	38.7 (32.9–44.4)
Visceral fat, cm^2^	85.1 (44.3–125.9)	80.1 (37.9–122.4)	58.9 (30.9–86.9)	56.5 (27.3–85.8)
Skeletal muscle ratio	1.000	1.078 (1.002–1.154)	0.926 (0.845–1.008)	0.954 (0.862–1.046)
Visceral fat ratio	1.000	0.971 (0.721–1.222)	0.795 (0.361–1.228)	0.795 (0.304–1.286)

* Skeletal muscle area at the third lumbar level;

^†^ Skeletal muscle index: skeletal muscle area/the square of the height, cm^2^/m^2^;

^§^ Visceral fat area at the third lumbar level.

### Survival according to the body composition ratio

**Fig 1:**
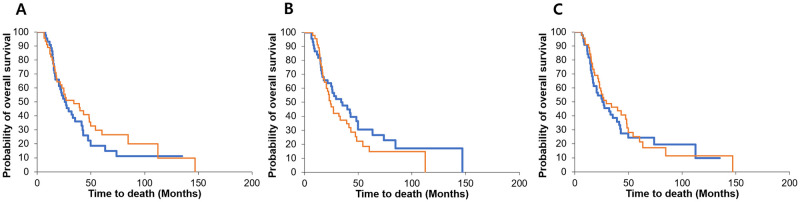
Survival according to the body mass index (BMI), skeletal muscle mass (SMM), and visceral fat mass (VFM) before resection. The Kaplan–Meier analysis found no significant difference in the overall survival (OS) according to the BMI, SMM, and VFM before resection. The orange and blue lines indicate the higher and lower groups, respectively. (A) BMI before resection (*P* = 0.311), (B) SMM before resection (*P* = 0.234), and (C) VFM before resection (*P* = 0.667).

The median follow-up was 26.9 months after resection. We examined the relationship between the OS and BMI as well as between the SMM and VFM before resection. The Kaplan–Meier analysis found no significant difference in the OS according to the BMI (*P* = 0.311), SMM (*P* = 0.234), and VFM (*P* = 0.667) before resection (Fig 1).

**Fig 2:**
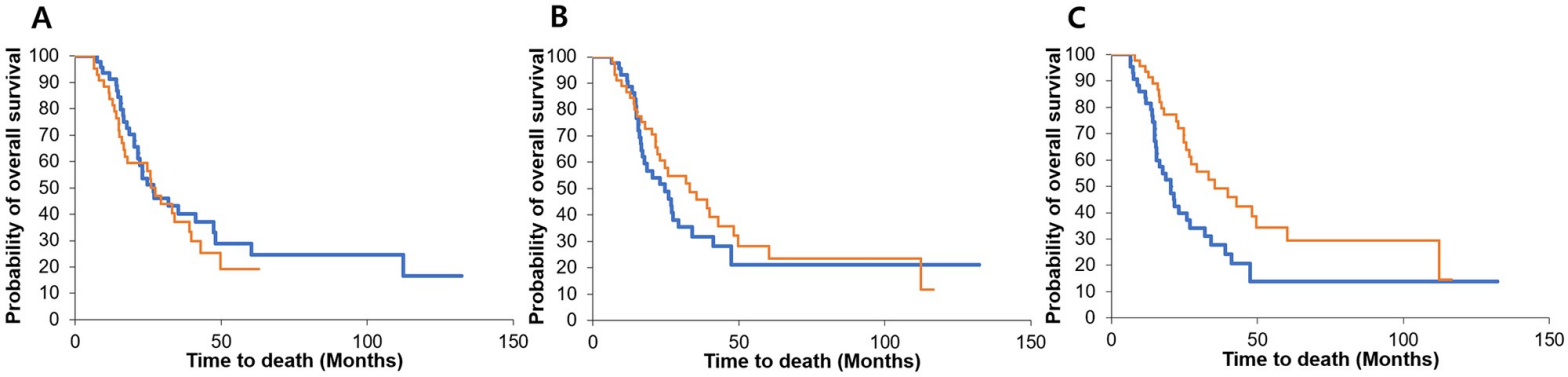
Survival according to the change in the skeletal muscle mass ratio (SMR). When we analyzed the SMR at each time point, there was no significant difference in the median overall survival (OS) between the higher and lower groups at 4 (*P* = 0.488) and 12 (*P* = 0.397) weeks after resection (A, B). However, the higher group showed a longer median OS than the lower group at 24 weeks (39 vs. 20 months, *P* = 0.008) (C).

**Fig 3:**
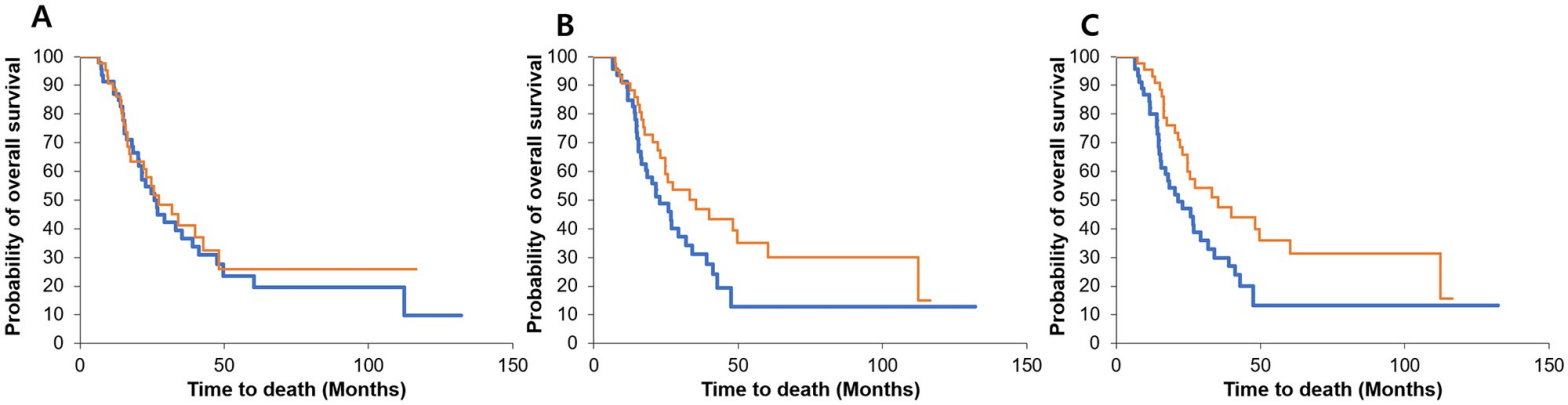
Survival according to the change in the visceral fat mass ratio (VFR). When we analyzed the VFR, the overall survival (OS) did not differ significantly between the higher and lower groups at 4 (*P* = 0.732) and 12 (*P* = 0.060) weeks after resection (A, B). However, the OS was longer in the higher visceral fat group at 24 weeks (35 vs. 22 months, *P* = 0.023) (C).

When we analyzed the SMR at each time point, there was no significant difference in the median OS between the higher and lower groups at 4 (*P* = 0.488) and 12 (*P* = 0.397) weeks after resection. However, the higher group showed a longer median OS than the lower group at 24 weeks (39 vs. 20 months, *P* = 0.008) (Fig 2). Similarly, when we analyzed the VFR, the OS did not differ significantly between the higher and lower groups at 4 (*P* = 0.732) and 12 (*P* = 0.060) weeks after resection. However, the OS was longer in the higher visceral fat group at 24 weeks (35 vs. 22 months, *P* = 0.023) (Fig 3). We analyzed the baseline characteristics of the higher and lower groups at 24 weeks and these are presented in S1 Table.

**Fig 4:**
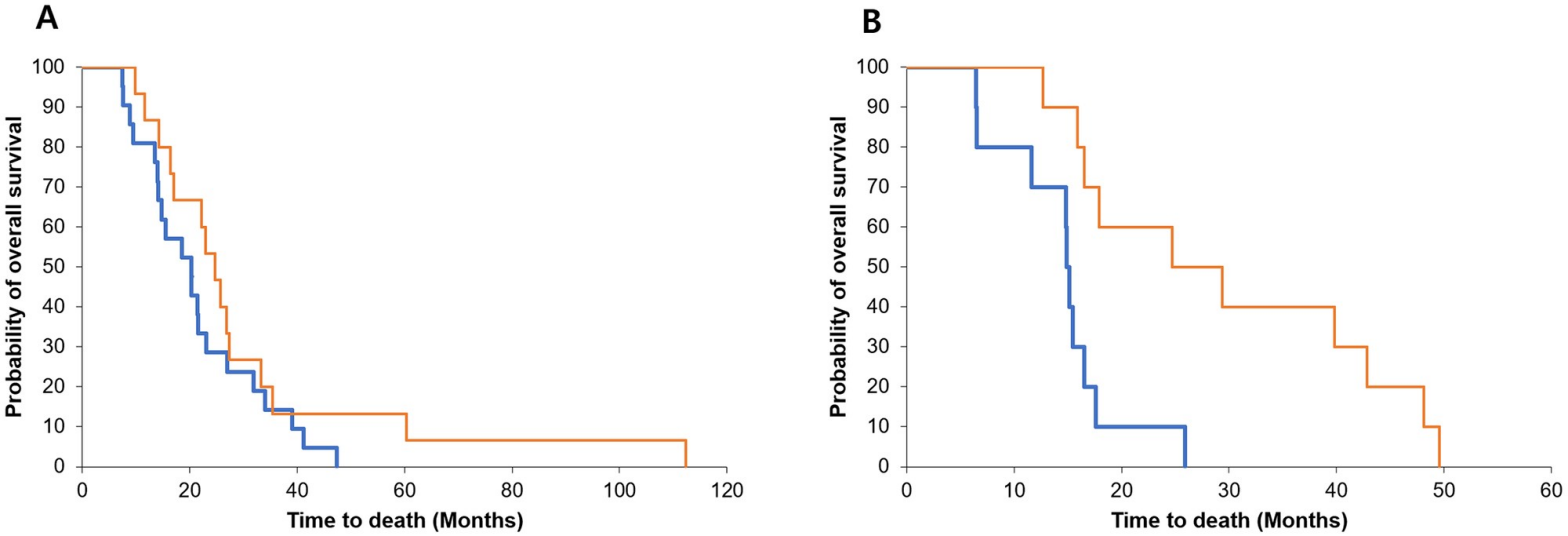
Survival according to the change in the body composition ratio in both gender groups at 24 weeks after resection. The patients were divided into the male group (A) and female group (B), and the effect of the restoration of skeletal muscle mass (SMM) at 24 weeks after surgery on the overall survival (OS) was analyzed using the Kaplan–Meier method. In this Kaplan–Meier analysis, the SMM and OS were not statistically significant in the male group (*P* = 0.213) but were significant in the female group (*P* = 0.002).

Patients were divided based on gender, and the effect of the restoration of SMM at 24 weeks after resection on the OS was analyzed using the Kaplan–Meier method. According to this analysis, the difference between SMR and OS was not significant in the male group (*P* = 0.213) but was significant in the female group (*P* = 0.002) (Fig 4).

A univariate analysis of prognostic factors for the OS was performed on several variables, including gender, age, adjuvant chemotherapy, adipopenia, and sarcopenia. As a result, sarcopenia and adipopenia were found to be significant prognostic factors. However, in the multivariate Cox regression analysis, only the SMR at 24 weeks (*P* = 0.023) was significantly associated with the OS, and the VFR at 24 weeks (*P* = 0.073) was not (Table 3).

**Table 3:** Prognostic factors for overall survival in the univariate and multivariate analyses at 6 months after resection of pancreatic cancer.

Factors	OS	Univariate analysis		Multivariate analysis	
	(month)	HR (95% CI)	*P* value	HR (95% CI)	*P* value
Sex (male)	26.5	1.35 (0.78–2.33)	0.289	1.13 (0.62–2.08)	0.683
Age (≥65)	27.6	1.16 (0.69–1.96)	0.585	1.04 (0.30–3.60)	0.947
Adjuvant chemotherapy	29.5	0.54 (0.28–1.00)	0.050	0.58 (0.29–1.12)	0.105
Adipopenia*	35.8	0.54 (0.32–0.92)	0.023	0.59 (0.33–1.05)	0.073
Sarcopenia†	35.8	0.49 (0.29–0.83)	0.008	0.52 (0.30–0.92)	0.023

* Adipopenia, patients whose ratio of visceral fat of the third lumbar area at 24 weeks point after resection to the visceral fat at the third lumbar area on pre-resection point is lower than the median value.

^†^ Sarcopenia, patients whose ratio of skeletal muscle of the third lumbar area at 24 weeks point after resection to skeletal muscle of the third lumbar area on pre-resection point is lower than the median value

### Survival in patients without early recurrence

**Fig 5:**
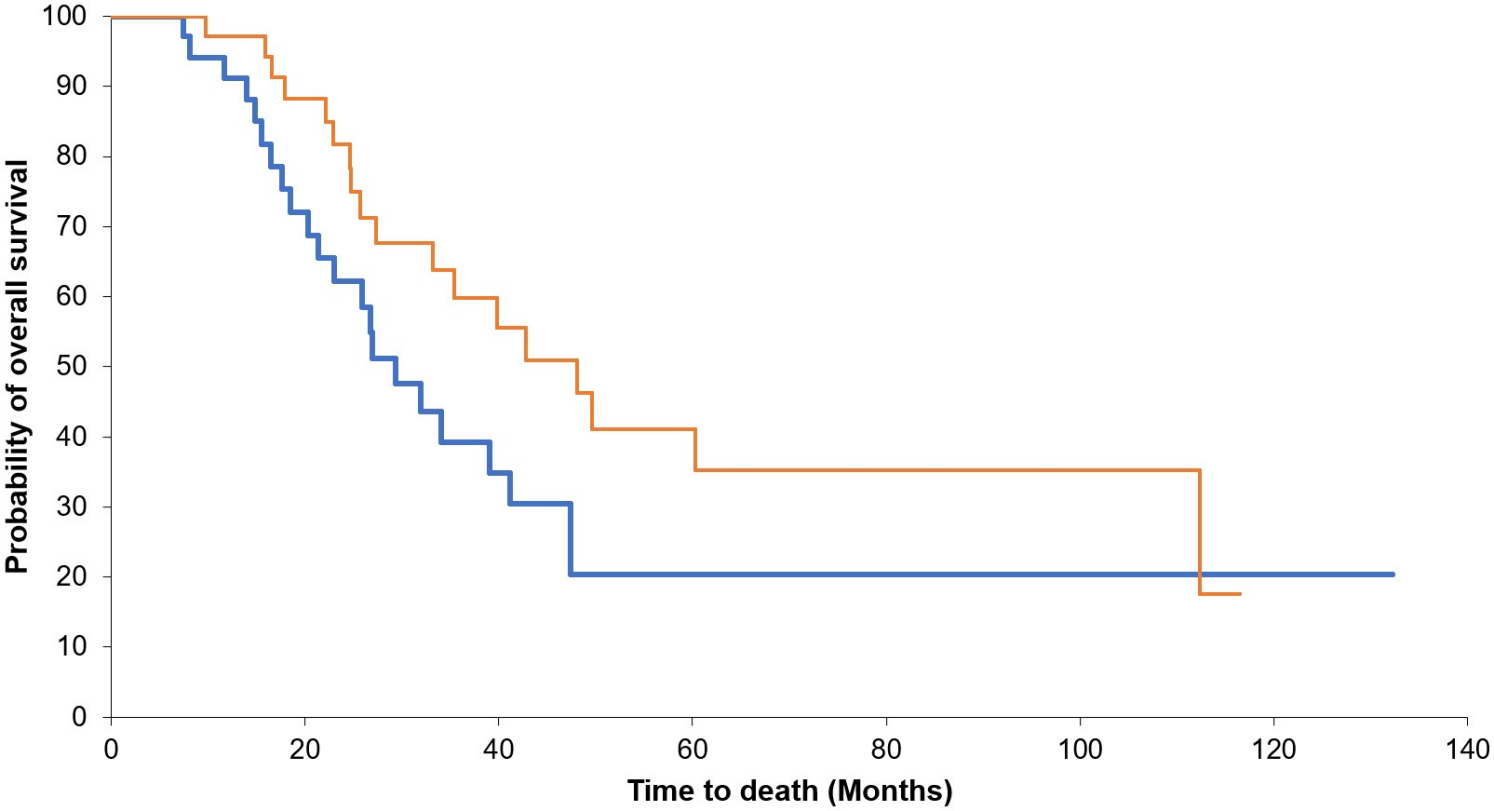
Subgroup analysis: Except for the early recurrence group. Overall survival (OS) according to the change in the skeletal muscle mass ratio (SMR) at 24 weeks after surgery. In the lower group (blue line), the SMR at 24 weeks after resection was related to shorter overall survival than that in the higher group (red line), but this was not statistically significant (*P* = 0.07).

We performed a subgroup analysis of 69 patients without recurrence at 6 months after resection (Fig 5). The higher group at the SMR at 24 weeks after resection was related to longer OS than the lower group (61.9 vs. 48.0 months) although this was not statistically significant (*P* = 0.07).

## Discussion

Several studies have suggested that sarcopenia at the time of diagnosis or preoperative weight loss may be associated with reduced overall survival in all stages of PC [8, 17, 21]. It may be natural for sarcopenia to reflect the nutritional status at the time of diagnosis [22], which can influence survival. At the time of diagnosis, sarcopenia cannot be manipulated; however, after resection, it can be controlled by cancer rehabilitation therapies, including nutritional support or medication [23]. Moreover, according to recent experimental models, an inhibition of the cachexia-related cytokine pathway may dramatically increase the lifespan by inhibiting cancer-induced weight loss although indicating minimal antitumor effects [24]. Therefore, it is worth investigating the serial changes in body composition that may influence survival in patients with resected PC.

In our research, there was no difference in the OS with respect to the SMM before resection. However, we found that a restoration of SMM at 24 weeks after resection is associated with favorable outcomes in patients with resected PC (39 vs. 20 months, *P* = 0.008). This may be because patients with depletion of SMM present higher chemotherapy-related toxicity and poor prognosis, whereas those with larger amounts of SMM may show fewer toxicities and better outcomes [25]. To the best of our knowledge, this is the first study analyzing the relationship between body composition trajectory and survival in patients with PC after resection. The data presented in this study could suggest that the preservation of muscle mass impacts survival and postoperative rehabilitation improves survival in patients with resected PC.

Patients with PC frequently experience recurrence even after complete resection followed by adjuvant treatment. To minimize the impact on sarcopenia by early recurrence, we performed a subgroup analysis, with the exception of early recurrence. The OS of patients with preserved SMM tended to be better. Currently, we are unable to provide an exact as to why the restoration of muscle mass was a more important factor with respect to survival than preoperative SMM in patients with resected PC, which is similar to the findings of a previous study that included patients with unresectable colorectal cancer [26].

A recent study reported that accelerated SMM and VFM losses were associated with reduced survival in patients with advanced PC [27]. In our study, the univariate analysis showed that the VFR and OS were related (*P* = 0.023). However, this was not significant in the multivariate Cox regression analysis (*P* = 0.073). Therefore, this result suggests that the changes in the SMM over time have a greater impact on survival than the VFR.

Recently, there have been some studies asserting that physical activity and exercise may have a positive effect on the management of cancer cachexia by improving health-related quality of life, physical performance [28, 29]. In a randomized, controlled trial of 231 patients with cancer, an 8-week physical exercise program showed significant improvement in patients’ physical performance [29]. Although there is insufficient evidence of its effectiveness and practicality to date, exercise appears to be beneficial to patients with or at risk of cancer cachexia [30, 31]. Accordingly, an exercise program for patients with cancer should be developed. Based on the result of our study, we believe that exercise from 12 to 24 weeks after PC resection is important for production of SMM; hence, it may be necessary to develop an exercise program during this post-resection period.

This study has some limitations. It was conducted at a single-institution, retrospective design with a small sample size. However, the strength of this study may be that we used an objective index of CT to measure the serial body composition changes with a duration of up to 6 months after resection. Although there is no consensus for defining sarcopenia in the Asian population, unlike the European population [11], our results may be applicable because the longitudinal body composition change can serve as a prognostic factor instead of the cross-sectional body composition.

In conclusion, this study suggests that the restoration of SMM at 24 weeks after surgery is an independent prognostic factor for survival in patients with resected PC. We propose that a development of therapeutic strategies focused on improving the OS is important for the rehabilitation and restoration of muscle mass in patients with resected PC.

## Summary

We found that the restoration of muscle mass at 24 weeks after resection is an independent prognostic factor for survival in patients with resected PC. The importance of this finding is that it can be helpful in the development of a therapeutic strategy for the rehabilitation of patients with PC after resection.

## Supporting information

S1 Table Characteristics of the higher and lower groups (at 24 weeks after resection). (DOCX)
