# Supplementary material for: Postoperative muscle mass restoration as a prognostic factor in patients with resected pancreatic cancer
Source: PLoS One. 2020 Sep 16;15(9):e0238649. doi: 10.1371/journal.pone.0238649 (PMC7494072; doi:10.1371/journal.pone.0238649)
Supplement: S1 Table — (DOCX) [file pone.0238649.s001.docx]

**S1 Table. Characteristics of the higher and lower groups (at 24 weeks after resection)**

| Variables | Higher group, n = 46 | Lower group, n = 43 | *P* |
| --- | --- | --- | --- |
| Median age (range), years | 62.5 (27–77) | 64 (35–84) | 0.761 |
| Sex, no (%) |  |  | 0.400 |
| Male | 26 (56.5%) | 29 (67.4%) |  |
| Female | 20 (43.5%) | 14 (32.6%) |  |
| BMI* (range) (kg/m^2^) | 21.5 (16.9–27.3) | 20.6 (15.4–30.9) | 0.238 |
| Diabetes mellitus | 24 (52.2%) | 14 (32.6%) | 0.098 |
| ASA grade† |  |  | 0.091 |
| I | 15 (32.6%) | 10 (23.3%) |  |
| II | 29 (63.0%) | 25 (58.1%) |  |
| III | 2 (4.4%) | 8 (18.6%) |  |
| Tumor location |  |  | 0.775 |
| Head | 29 (63.0%) | 28 (65.1%) |  |
| Body | 7 (15.22%) | 8 (18.6%) |  |
| Tail | 10 (21.74%) | 7 (16.3%) |  |
| Differentiation of tumor |  |  | 0.374 |
| Well differentiated | 5 (10.9%) | 2 (4.7%) |  |
| Moderately differentiated | 34 (73.9%) | 37 (86.1%) |  |
| Poorly differentiated | 3 (6.5%) | 3 (7.0%) |  |
| Undifferentiated | 4 (8.7%) | 1 (2.3%) |  |
| Type of resection |  |  | 0.423 |
| Standard PD | 6 (13.0%) | 10 (23.3%) |  |
| Pylorus-preserved PD | 26 (56.5%) | 20 (46.5%) |  |
| Distal pancreatectomy | 14 (30.4%) | 13 (30.4%) |  |
| Surgical margin state |  |  | 0.017 |
| R0 | 40 (87.0%) | 27 (62.8%) |  |
| R1 | 6 (13.0%) | 16 (37.2%) |  |
| Adjuvant chemotherapy (≥4 cycles) | 38 (82.6%) | 37 (86.0%) | 0.878 |

* BMI, body mass index at the time of diagnosis; † ASA grade, American Society of Anesthesiologists grade; ‡ PD, pancreaticoduodenectomy
